# The Common Task

**Published:** 1907-12-28

**Authors:** 


### THE COMMON TASK.

Correspondence and Queries for this section should be sent to the Editor of The Hospital, 28 Southampton Street, Strand, London, and marked "Nursing Administration."

### An Eight Hours' Day.

One of the main problems in arranging hospital hours of duty is that while twelve hours on duty seems certainly too long—eight hours is hardly long enough. No one can be on duty continuously for even eight hours, so that two breaks, or at least one meal-time, must be deducted. This reduces the time actually spent with the patients to little over seven hours, and we are not surprised that nurses complain that they do not get a real grip of the cases placed in their charge when the time for observation in each day is so short. The solution of the difficulty would appear to lie not with making three shifts in the twenty-four hours, but in so arranging matters that a reasonable period of leisure and recreation is afforded to the nurses in the midst of their duties. The excessive fatigue often experienced by nurses, both in hospital and out of it, would be lessened to a marked extent if exact punctuality provided for their getting the time off which is marked down for them. Meals ought to be enjoyed in comfort, so that nurses return to the wards thoroughly rested, instead of languid with the effort of trying to digest food bolted at lightning speed. Then the ten hours actually spent on duty would not be found excessive.

### English Nurses in America.

It is quite extraordinary that nurses still believe the American public to be sitting down waiting for Englishwomen to come and nurse them. Occasionally, it is true, a nurse is chosen from some world-famous English training school to take charge of an American hospital where English methods are admired. But the American training schools are turning out year by year more than sufficient nurses for the needs of the public, and the competition for the most highly-paid work in the cities is fully as great as in London. Nothing but disaster can await the nurse who starts off for New York confident of speedily earning a fortune, and expecting to be preferred before the highly-trained nurses of the country, who can be personally recommended by matrons in a position to introduce them to work. We have only known one nurse who succeeded in making a living over the seas, and she had to go as far as California. Matrons would do well to warn nurses when they leave the hospital to take up private work that no country affords better prospects for private nursing than their own.

### A Good Example.

The London County Council is doing good service beyond its own immediate sphere by offering scholarships for women desiring to take up the calling of midwife. In London, it is true, midwifery, if anywhere, may hope to be self-supporting. But the prizes it offers are not sufficient to tempt into the ranks all who are wanted. It is to be hoped that other County Councils less well situated with regard to the supply of pupil midwives will follow the example set by London, and stimulate the numbers in the calling by the offer of assistance in training. But, after all, what is urgently needed is additional centres for the training of midwives. A vast deal of material is wasted in Poor-law Infirmaries which might, with but little expense, be turned to account. More, again, is wasted in the numerous large towns which can boast no training school for midwives. Indeed, the number of training schools for this purpose is ridiculously out of proportion to the needs of the country, and until there is some forward movement in this direction the shortage of midwives cannot be repaired. We doubt indeed if the number of midwives in training is much in excess of the number annually falling out on account of ill-health and superannuation. There are eighteen training school centres licensed by the Central Midwives Board in London, twenty-nine for the rest of England, two in Wales, six in Scotland, and seven in Ireland. Were the number of the provincial centres doubled it would still be far too small to meet the demands likely to be made during the next few years.

### Holt Ockley.

The name is given to the system which introduces village women to the principles and practice of cleanliness, instructs them in first aid, and employs them as "cottage resident nurses" in the homes of the rural poor. The promoters of the scheme have shown singular judgment in their choice of women to undergo the brief period of training prescribed, and in parts of Oxfordshire and in other counties the plan has worked satisfactorily. It is said to raise the tone of the patients to have one of their own class living with them, and doing all the work of the cottage for several days or weeks, as the case may be, and no doubt improved methods of performing the daily duties which fall to these hard-worked and ignorant mothers are often taught through this means, and could hardly in any other way be so readily acquired. But it would seem that "Holt Ockley" is also of the nature of a creed, and like some other creeds the leading note among its most ardent supporters appears to be intolerance. At a representative meeting on nursing held recently in Oxford, one of these ladies committed herself to the statement that "trained nurses could do nothing but harm among the very poor in rural districts," and that "it was essential for the sick nurse to undertake the entire work of the cottage." The theories which inspire such sentiments are based on an absolute misconception of the trained nurse's duties. There are many reasons outside the pressing one of fit accommodation which render it undesirable that the district nurse should reside in the cottage of her patients. The higher the training of the nurse the more ready she will always be found to perform any kind of work which will conduce to her patient's recovery, but to distract her attention from the care of the sick to that as charwoman to the relatives is to confuse two entirely different "metiers." It will be interesting to see whether "Holt Ockley" survives the onward march of events in village nursing.

